# Underreporting of workers’ injuries or illnesses and contributing factors: a systematic review

**DOI:** 10.1186/s12889-023-15487-0

**Published:** 2023-03-24

**Authors:** MinJung Kyung, Soo-Jeong Lee, Caroline Dancu, OiSaeng Hong

**Affiliations:** Department of Community Health Systems, School of Nursing, University of California San Francisco, 2 Koret Way, Suite #N-505, San Francisco, CA 94143 USA

**Keywords:** Mandatory reporting, Underreporting, Occupational accident, Work-related illness, Workers’ compensation

## Abstract

**Background:**

Accurate identification of work-related health problems is important to understand workplace safety issues and develop appropriate interventions. Although workers’ reporting of work-related injuries or illnesses is the very first step of the reporting process, many workers may encounter challenges in reporting them to their management or workers’ compensation (WC) programs. This systematic review aimed to identify the level of workers’ underreporting of work-related injuries and illnesses and the contributing factors and reasons for underreporting among US workers.

**Methods:**

This study searched PubMed (Medline), PsycINFO (ProQuest), CINAHL (EBSCOhost), EMBASE (Embase.com), and Social Science Citation Index (Web of Science) using search terms related to underreporting of work-related injury or illness.

**Results:**

Twenty studies (17 quantitative and three mixed methods studies) were identified. The studies investigated reporting to management (*n* = 12), WC programs (*n* = 6), multiple organizations (*n* = 1), and not specified (*n* = 1). The timeframe used to measure reporting prevalence varied from three months to entire careers of workers, with the most common timeframe of 12 months. This review indicated that 20–91% of workers did not report their injuries or illnesses to management or WC programs. From quantitative studies, contributing factors for injury or illness underreporting were categorized as follows: injury type and severity, sociodemographic factors (e.g., age, gender, education, and race/ethnicity), general health and functioning, worker’s knowledge on reporting, job and employment characteristics (e.g., work hour, job tenure, work shift, type of occupation, and physical demand), psychosocial work environment (e.g., supervisor support, coworker support, and safety climate), and health care provider factors. From the review of qualitative studies, the reasons for underreporting included the following: fear or concern, cumbersome time and effort in the reporting process, lack of knowledge regarding reporting, perceptions of injuries as not severe or part of the job, and distrust of reporting consequences.

**Conclusions:**

The review findings indicated that low wage earners, racial/ethnic minority workers, and workers who perceive a poor psychosocial work environment encounter more barriers to reporting a work-related injury or illness. This review also identified variations in the measurement of work-related injury reporting across studies and a lack of standardized measurement.

**Trial registration:**

The review was registered in the PROSPERO, an international database of prospectively registered systematic reviews in health and social care (CRD42021284685).

**Supplementary Information:**

The online version contains supplementary material available at 10.1186/s12889-023-15487-0.

## Background

Accurate identification of work-related injuries or illnesses is an important action to understand workplace health and safety problems and develop an effective prevention program [1]. Identifying workplace health and safety problems early helps companies design and implement preventative strategies before the problems become more significant and prevalent [2]. For injured workers, workers’ compensation (WC) programs support them to receive timely health care, prevent long-term disability, and mitigate financial losses by providing wage replacement for lost workdays [3]. Thus, underreporting of work-related injury or illness has important consequences for both employers and workers.

The measurement of occupational injuries and illnesses ultimately depends on workers’ reporting behavior [4]. The United States Occupational Safety and Health Administration (OSHA) provides the legal foundation of employee’s rights to report injuries free from retaliation and prohibits employers from taking any adverse actions against employees for the reporting [5]. According to the OSHA’s 2014 updated reporting guidelines, employers are required to report all work-related fatalities within eight hours and all-in patient hospitalizations, amputations, and losses of an eye within 24 h of finding out about the incident [5]. Despite the basic rights, researchers have indicated that many workers encounter challenges in reporting a work-related injury or illness to their supervisor or company official [6, 7]. Scherzer et al. [8] found that among workers who had pain or discomfort during the previous 12 months, only 33% reported their symptoms to company officials and 26% filed a WC claim. Further, there have been changes in the nature of work including the demographic diversity of workers and non-standard work arrangements such as short-term contracts or outsourcing of function in organizations. With these changes, more workers are subject to precarious employment which can result in reluctance to report their injury to a company or file a WC claim [9].

Workers’ reporting of work-related injury or illness can be affected by various factors and an evaluation of underreporting may contribute to improvement of the reporting environment and system. Menzel [10] reviewed underreporting of work-related injuries or illnesses to OSHA but reporting behaviors in this review were not limited to workers. Since documentations of work-related injuries and illnesses involve multiple steps, identifying the filters in each reporting step is important to develop and implement targeted interventions. To date, there has not been a systematic review of workers’ injury reporting behaviors, which is the first step of the reporting process. Therefore, we conducted a systematic review of the literature to determine the prevalence of worker-level underreporting, factors contributing to their underreporting and why workers do not report work-related injuries or illnesses.

## Methods

### Eligibility criteria

Studies were eligible for inclusion if they investigated workers’ reporting behaviors to company officials or WC programs. In our review, a worker was defined as a person employed for wages or salary including apprentices. As the initial search located only a small number of studies that assessed contributing factors or reasons for reporting work-related injuries or illnesses, no restriction was placed on the publication date. Quantitative, qualitative, and mixed method studies were included in this review to achieve an adequate depth of understanding. Quantitative studies included randomized controlled trials (RCTs), non-randomized studies, and descriptive studies. Quantitative studies provided data on the magnitude of underreporting and the characteristics of workers who were more likely to underreport an occupational injury or illness. Qualitative studies were included to identify more detailed information regarding why workers did not report a work-related injury or illness. As WC programs and reporting processes vary by country, this review only included studies conducted in the United States and written in English. Because the scope of this review was identification of factors and reasons associated with workers’ reporting behavior, studies were excluded if they examined underreporting at the level of employers or physicians, such as reporting to OSHA or reporting to WC by physicians or employers.

### Information sources and search strategy

The following five databases were searched in collaboration with a librarian: PubMed (Medline), PsycINFO (ProQuest), CINAHL (EBSCOhost), EMBASE (Embase.com), and Social Science Citation Index (Web of Science). The last search was conducted on November 15, 2022. Multiple search terms were customized and applied to each database, including mandatory reporting, underreporting, underestimating, occupational injuries, occupational accidents, occupational diseases, and work-related illnesses. The search strategy is detailed in Appendix 1. Medical Subject Headings (MeSH) and text words were applied where it was appropriate. Searches were supplemented by hand-searching the reference lists of articles identified from initial database searching to locate additional relevant articles.

### Selection and data collection process

Retrieved articles from each database and reference search were imported into Covidence software (Covidence online review manager 2021, www.covidence.org). Duplicated records were identified and removed. The titles and abstracts of all the citations were independently screened by two reviewers (MK and CD). The remaining relevant articles were retrieved for full-text review to determine whether the studies met the eligibility criteria. For disagreements, the two reviewers discussed until they reached a consensus regarding inclusion or exclusion.

### Data items

#### Descriptive data

The following descriptive data were extracted from each study: authors, publication year, study design, measures (e.g., questionnaire, interview, focus group, and administrative data), sampling method, sample size, gender, race/ethnicity, mean age, and workers and workplace setting.

#### Reporting behavior

As the outcomes of this review, reporting behavior was reviewed for the prevalence of not reporting, work-related injury or illness, contributing factors to reporting of work-related injury/illness, and reasons for not reporting. We also examined the type of reporting (e.g., reporting to management and WC filing) and type of injury/illness (e.g., any work-related injury or illness, musculoskeletal pain, sharp injury, etc.).

### Study risk of bias assessment

The risk of bias of included studies was appraised by the two reviewers who are enrolled in a PhD program, using the Mixed Methods Appraisal Tool (MMAT), a valid and reliable measure of systematic review of mixed method studies [11, 12]. The MMAT includes criteria for five study designs: qualitative research, RCTs, non-randomized controlled trials, quantitative descriptive studies, and mixed method studies. For ensuring appropriateness of using the tool, the MMAT comprises screening questions for all types of studies and a checklist for each study design. The latest version of MMAT [11] comprises five criteria for evaluating each study type; the scoring ranges from 1 to 5 with a higher score indicating better quality (5 = high, 3–4 = moderate, < 3 = low). In this review, all included studies were evaluated using screening questions prior to applying the tool. Further details on the quality appraisal can be found in Supplemental Appendix 2.

#### Effect measures and synthesis methods

Table 1 presents a summary of characteristics of the extracted studies. The review summary on the prevalence of no reporting, contributing factors, and reasons for not reporting of work-related injuries or illnesses are presented in Table 2. From quantitative studies, contributing factors to reporting of work-related injuries or illnesses were categorized with similar properties and summarized with various measures of association including odds ratio (OR), beta coefficient, and prevalence rate (PR). From qualitative studies, reasons for not reporting work-related injuries or illnesses were analyzed in three stages using a thematic synthesis [13]. First, all texts were coded inductively. Second, codes were categorized by similarity to organize descriptive themes. Third, in an interpretation stage, analytical themes were generated.

**Table 1:** Characteristics of included studies

Author, Year	Study design, Measure	Sampling method	Sample characteristics		Worker and workplace setting	Quality appraisal, Certainty
			Size	Gender, Race/ethnicity, Mean age		
Weddle, 1996 [4]	CS, Q	Convenient	368	F: 56% M:44% Not provided Mean 39 years	Workers in the environmental service department of 5 hospitals in Baltimore	2, Low
Haiduven, 1999 [14]	CS, Q	Convenient	549	Not provided	Healthcare workers in a public teaching hospital in Santa Clara	3, Moderate
Rosenman, 2000 [15]	CS, Q and I	Random	1,598	F: 41% M:59% W: 67% B:28% Not provided	General workers in Michigan	5, High
Biddle, 2003 [16]	PC I and AD	Random	1^st^ wave: 1,598 2^nd^ wave: 1,118	Not provided	General workers in Michigan	4, Moderate
Scherzer, 2005 [8]	CS, Q	Convenient	941	F: 99% M:1% H: 76% Mean 42 years	Las Vegas hotel room cleaners	4, Moderate
Fan, 2006 [17]	CS, Q	Random	321	F: 47% M: 53% W:88% Not provided	General workers in Washington, DC	5, High
Siddharthan, 2006 [18]	CS (MM), Q and F	Convenient	Q: 15,319 F: 28	F: 84% M:16% W:70% B:17% Mean 50-59 years	Workers at veteran administration hospitals in Washington, DC	2, Low
Gershon, 2007 [19]	CS, Q	Random	1,156	F: 87% M:13% Not provided Mean 49 years	Unionized registered nurses (RNs) employed in a wide range of non-hospital settings in New York	4, Moderate
Makary, 2007 [20]	CS, Q	Convenient	699	F: 31% M: 69% Not provided Not provided	Surgeons in training at residency programs in general surgery certified by the Accreditation Council for Graduation Medical Education	4, Moderate
Donnelly, 2013 [21]	CS, Q	Convenient	336	Not provided	Residents, fellows, and practicing dermatologists	3, Moderate
Lipscomb, 2013 [6]	CS, Q	Convenient	1,020	Not provided Not provided Mean 27 years	Carpenter apprentices in 3 union training programs in Chicago, Illinois, St. Louis	4, Moderate
Moore, 2013 [22]	CS (MM), Q and F	Convenient	135	F:1% M:99% W: 92% Mean 45 years	Union construction workers in Northwest	2, Low
Qin, 2014 [23]	CS, Q	Convenient	2,639	F:90% M:10% W: 50% B: 37% Not provided	Nursing homes workers in Maine, Maryland, Massachusetts, and Rhode Island	4, Moderate
Boden, 2015 [24]	CS, Q and AD	Convenient	1,572	F:91% M:9% W: 77% Not provided	Patient care workers	3, Moderate
Pompeii, 2016 [25]	CS Q and F	Convenient	5,385	F: 72% M:28% W: 45% B: 23% A: 9% H: 7% Not provided	All workers in 6 hospitals in Texas and North Carolina who were likely to interact with patients and/or visitors as part of their job	4, Moderate
Deipolyi, 2017 [26]	CS, Q	Random	908	F:10% M:90% Not provided Mean 45 years	Interventional radiologist members of the Society of Interventional Radiology	4, Moderate
Green, 2019 [27]	RCT, Q	Random	390	F:55% M:45% W: 30% H:68% Not provided	Full-time janitors in the Service Employees International Union	4, Moderate
Yang, 2019 [28]	CS, Q	Random	7,395	F:38% M:61% Not provided Not provided	Residents taking the 2017 American Board of Surgery In Training Examination	5, High
Anderson, 2021 [29]	CS, Q and I	Convenient	620	F:57% M:43% W:57% H:13% Mean 45 years	Janitors and custodians who are currently employed or had been employed in the past year	3, Moderate
Lee, 2021 [7]	CS, Q and I	Convenient	171	F: 58% M:42% A:67% H:20% Mean 48 years	Cleaning workers in Northern California	4, Moderate

*CS* Cross sectional, *PC* Prospective cohort, *RCT* Randomized controlled trial, *MM* Mixed method, *Q* Questionnaire, *I* Interview, *AD* Administrative data, *F* Focus group, *F* Female, *M* Male, *W* White, *B* Black, *H* Hispanic, *A* Asian

**Table 2:** Prevalence, contributing factors, and reasons for reporting of work-related injuries or illnesses

Author, Year	Type of reporting	Type of injury/illness	Prevalence of not reporting	Contributing factors to injury/illness reporting	Reasons for not reporting
Weddle, 1996 [4]	Reporting to management	All injuries including needlesticks, cleaning chemical burns and eye splashes, back pain after heavy lifting, and any others for 12 months	39%		❿ Fear ❿ Cumbersome time and effort ❿ Perceptions of injuries as not severe or a part of the job
Haiduven, 1999 [14]	Reporting to management	All percutaneous/mucocutaneous injuries in the last 5 years	47%		❿ Cumbersome time and effort ❿ Perceptions of injuries as not severe or part of the job ❿ Distrust of reporting
Rosenman, 2000 [15]	WC filing	1.Diagnosed repetitive trauma with neck, upper extremity, and low back work-related musculoskeletal disease during the 12-week 2. Resulting in missed more than 7 consecutive days of work during the 12-week	1. 74% 2. 25%	*Facilitators* ❿ Sociodemographic factors : Lower annual income ❿ Job and employment : Increased length of employment ❿ Injury type and severity : Restriction on activity : Off from work 7 days or more ❿ Psychosocial work environment : Workers’ dissatisfaction with Coworkers ❿ Health care providers : A provider not belonging to the company	
Biddle, 2003 [16]	WC filing	1.Work-related pain in backs, wrists, hands, or shoulders (repetitive trauma) identified by physicians in the past 12 months 2. Resulting in lost work time 3. Resulting in missed more than 7 consecutive days of work	1. 68% 2. 45% 3. 27%	*Facilitators* ❿ Sociodemographic factors : Older age ❿ Injury type and severity : Severe wrist condition ❿ Job and employment : Higher physical exertion required *Barriers* ❿ General health and functioning : Better role functioning : Better current health	
Scherzer, 2005 [8]	Reporting to management, WC filing	Any pain or discomfort during the past 12 months that he/she feels might have been caused or made worse by one's work as a hotel room cleaner	Management: 67% WC: 74%		❿ Fear ❿ Cumbersome time and effort ❿ Perceptions of injuries as not severe or part of the job ❿ Lack of knowledge
Fan, 2006 [17]	WC filing	Positive response to at least one of the following: • In the past 12 months, have you been injured while performing your job? • Has a doctor or other medical professional told you that you have a work-related illness?	47%	*Facilitators* ❿ Sociodemographic factors : Being married ❿ Job and employment : Service occupation : Precision, craft, repair occupation : Operators, fabricators, laborers ❿ General health and functioning : Obesity	
Siddharthan, 2006 [18]	Not specified	Work-related musculoskeletal pain in the last 12 months that require to reschedule work	35%	*Facilitators* ❿ Sociodemographic factors : Age 50 years or older : Hispanic workers *Barriers* ❿ Injury type and severity : More than 3 injuries in the last 12 months ❿ Job and employment : Job tenure 5 years or more : Evening or Night shift : Work more than 80 h in 2 weeks ❿ Psychosocial work environment : Worker’s safety as a priority of management	❿ Fear ❿ Cumbersome time and effort ❿ Perceptions of injuries as not severe or part of the job ❿ Lack of knowledge
Gershon, 2007 [19]	Reporting to management	Exposure to blood and body fluids in the last 12 months	49%		❿ Fear ❿ Cumbersome time and effort ❿ Lack of knowledge ❿ Distrust of reporting
Makary, 2007 [20]	Reporting to management	Needlestick injuries in the past 5 years	51%	*Barriers* ❿ Sociodemographic factors : Male workers ❿ Injury type and severity : Injury not involving a high-risk patient : Total number of needlesticks during training ❿ Job and employment : Occurrence in operating room ❿ Knowledge on reporting : No information from other person	
Donnelly, 2013 [21]	Reporting to management	Sharp injuries in the past 12 months	65%		❿ Fear ❿ Cumbersome time and effort ❿ Perceptions of injuries as not severe or part of the job ❿ Lack of knowledge ❿ Distrust of reporting
Lipscomb, 2013 [6]	Reporting to management	Any work-related injury or illness (entire life)	20%	*Facilitators* ❿ Psychosocial work environment : Safety incentive for not having an injury : Coaching for unsafe behaviors *Barriers* ❿ Psychosocial work environment : Discipline behaviors for the injured	
Moore, 2013 [22]	Reporting to management	Any work-related injury or illness (entire life)	27%		❿ Fear ❿ Cumbersome time and effort ❿ Perceptions of injuries as not severe or part of the job ❿ Lack of knowledge ❿ Distrust of reporting
Qin, 2014 [23]	WC filing	Low back pain in the past 3 months at least once	91%	*Facilitators* ❿ Injury type and severity : Severe pain ❿ Job and employment : Higher physical demand : Higher job strain : Higher social support *Barriers* ❿ Sociodemographic factors : Higher education level	
Boden, 2015 [24]	Reporting to management	Sharp injuries during the past 12 months	21%	*Facilitators* ❿ Sociodemographic factors : Female workers ❿ Psychosocial work environment : Safety practice scale	
Pompeii, 2015 [25]	Reporting to management	Type II violence (patient/visitor-on-worker) in the past 12 months	51%	*Facilitators* ❿ Injury type and severity : involving in serious injury such as Physical assault or threat ❿ Job and employment : Nurse : Nurses’ aid : Security guard	❿ Fear ❿ Cumbersome time and effort ❿ Perceptions of injuries as not severe or part of the job ❿ Lack of knowledge ❿ Distrust of reporting
Deipolyi, 2017 [26]	Reporting to management	Needlestick injuries in the past 5 years	33%		❿ Fear ❿ Cumbersome time and effort ❿ Perceptions of injuries as not severe or part of the job ❿ Lack of knowledge ❿ Distrust of reporting
Green, 2019 [27]	WC filing	Any work-related injury or illness in the past 6 months	84%	*Facilitators* ❿ Psychosocial work environment : Safety training	❿ Fear ❿ Cumbersome time and effort ❿ Perceptions of injuries as not severe or part of the job ❿ Lack of knowledge
Yang, 2019 [28]	Reporting to management	Needlestick injuries in the past 6 months	29%	*Facilitators* ❿ Sociodemographic factors : Male workers ❿ Job and employment : Had less than 8 h off and between shift 3 times or more : Worked more than 80 h 3 times or more in a week ❿ General health and functioning : Poor general health status	❿ Fear ❿ Cumbersome time and effort ❿ Perceptions of injuries as not severe or part of the job
Anderson, 2021 [29]	WC filing	Any work-related injury or illness in the past 12 months	55%		❿ Fear ❿ Lack of knowledge
Lee, 2021 [7]	Reporting to management	Chemical-related symptoms in respiratory, eye, skin, neurological, and gastrointestinal systems (e.g., cough, burning in nose or throat, itchy or burning eyes, rash, headache, and nausea) in the past 12 months	74%	*Facilitators* ❿ Sociodemographic factors : College education *Barriers* ❿ Sociodemographic factors : Female workers : Asian workers	

*WC* Workers’ compensation

#### Reporting bias and certainty assessment

The overall certainty of the evidence was determined by a single reviewer (MK) using two separate tools. For quantitative evidence, the Grading of Recommendations, Assessment, Development and Evaluation (GRADE) approach was used [30]. The certainty of evidence was defined as high, moderate, low, or very low by considering potential limitations due to risk of bias, inconsistency, indirectness of results, imprecision, and publication bias for each outcome [30]. For mixed methods studies, the certainty of evidence was examined using the criteria of support by Bray et al. [31]. This criteria consisted of truth value/bias, explanation credibility, weakness minimization, consistency between inside and outside view, and publication bias using five assessment levels: strong, moderate, low, very low, and inconsistent [31].

## Results

### Study selection

**Fig. 1:**
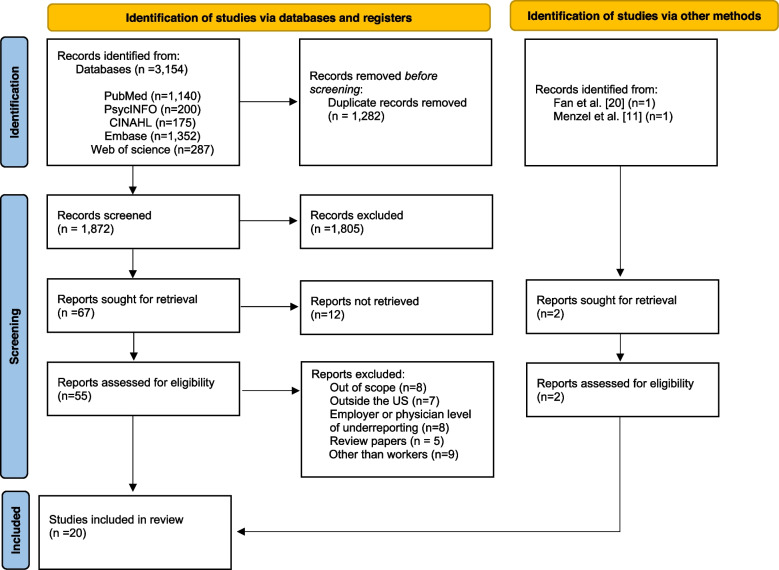
PRISMA flow diagram of the screening

The literature search yielded 1,872 unique references, of which 1,805 records were excluded after screening of the titles and abstracts. After full texts of the remaining 55 articles were reviewed, 37 were excluded because they did not meet the eligibility criteria as described in Fig. 1. An additional two articles were identified from searches of publication citations, resulting in 20 studies for the final synthesis. The study selection process is illustrated in Fig. 1 with reasons for exclusion.

### Study characteristics

Among the 20 studies included in this review, 17 used quantitative study designs and three used mixed methods (Table 1). Almost all studies (*n* = 18) used a cross-sectional design. One study used a prospective cohort design and another study used an RCT. For data collection, 12 studies used questionnaires or interviews; eight studies used two or more data collection methods such as questionnaire, interview, administrative data, or focus group. Thirteen studies had convenience samples and seven had random samples. The sample size of included studies ranged from 135 to 15,319. The percentage of females ranged from 1 to 99% and the percentage of Whites ranged from 30 to 92%. The mean age of study participants ranged from 27 to 59 years. The study samples included various workers such as healthcare workers, cleaning staff, carpenters, construction workers, and radiologists.

### Risk of bias in studies

Review of the risk of bias of each study is presented in Table 1. The overall quality of the RCT study (*n* = 1) was moderate due primarily to an unclear description of double-blinding [27]. Among nine non-randomized studies, the risk of bias was high for three studies and moderate for six studies because of non-representative samples and no control of potential confounders [6, 7, 15–17, 20, 23, 24, 28]. All descriptive quantitative studies used clearly defined measures of injury or illness underreporting and six studies used a convenience sampling method resulting in a limited generalizability [4, 8, 14, 19, 21, 26, 29]. Five studies reported response rates ranging from 26 to 49% or did not report a response rate [14, 19, 21, 26, 29]. The risk of bias in the three mixed method studies was poor to moderate [18, 22, 25]. Siddharthan et al. [18] adequately described the rationale for their study, but the divergences and inconsistencies between the quantitative and qualitative findings were not fully addressed and the integration of results was considered ineffective. Moore et al. [22] also did not explain the integration of qualitative and quantitative results and divergences and inconsistencies between them. The moderate quality of the study by Pompeii et al. [25] resulted from a lack of a rationale for the study and a limited description of divergences and inconsistencies between qualitative and quantitative results.

### Results of individual studies

Measurement methods and the prevalence of underreporting of work-related injuries or illnesses are presented in Table 2. Among the included studies, six studies measured underreporting of work-related injuries or illnesses to WC programs [15–17, 23, 27, 29], 12 studies measured underreporting to management [4, 6, 7, 14, 19–22, 24–26, 28], and one study measured underreporting to both (WC programs and management) [8]. The remaining study did not specify the entity of reporting [18]. The timeframe used to measure the prevalence of underreporting varied from three months (*n* = 2) [15, 23], six months (*n* = 2) [27, 28], 12 months (*n* = 11) [4, 7, 8, 16–19, 21, 24, 25, 29], five years (*n* = 3) [14, 20, 26], to across entire careers (*n* = 2) [6, 22]. The types of reported injury or illness included injury from sharps or exposure to blood or body fluid (*n* = 7) [14, 19–21, 24, 26, 28], musculoskeletal injury or illness (n = 4) [15, 16, 18, 23], workplace violence (*n* = 1) [25] and chemical-related symptom (*n* = 1) [7]. The measurement of reporting to WC programs also varied by study. Three studies [15–17] used conservative definitions of injury or illness reporting such as injury or illness resulting in missed work for more than seven consecutive days or a diagnosed work-related disease.

### Results of syntheses

Overall, workers’ underreporting prevalence ranged from 20 to 91%. Specifically, the prevalence of underreporting to management ranged from 20 to 74% [4, 6–8, 14, 19–22, 24–26, 28] and workers’ underreporting to WC programs ranged from 25 to 91% [8, 15–17, 23, 27, 29]. The risk of bias of the included studies was overall moderate. As presented in Table 2, 12 studies examined the association between various factors and reporting of work-related injuries or illnesses [6, 7, 15–18, 20, 23–25, 27, 28]. Those contributing factors were grouped into seven categories: injury type and severity, sociodemographic factors, general health and functioning, worker’s knowledge regarding reporting, job and employment characteristics, psychosocial work environment, and healthcare provider. Twelve studies investigated the reasons for workers’ underreporting of their injury or illness [4, 8, 14, 18, 19, 21, 22, 25–29]. Five overarching themes were derived from the thematic synthesis of the reasons similar in nature: (1) fear; (2) cumbersome time and effort in reporting process; (3) lack of knowledge regarding reporting; (4) perceptions of injuries as not severe or part of the job; (5) distrust of reporting process.

#### Contributing factors

##### Injury type and severity

Six studies [15, 16, 18, 20, 23, 25] identified injury type and severity as a significant contributing factor to injury or illness reporting. There was a positive association between higher severity and injury or illness reporting to WC [15, 16, 23]. Workers with needlestick injuries involving a high-risk patient such as HIV, hepatitis B, hepatitis C, or injection drugs were more likely to report their injury or illness to management than those with injuries not involving a high-risk patient [20] and workers experiencing serious injury were more likely to report the injury to management [25]. Workers who had more than three work-related injuries in the past 12 months [18] were less likely to report their injury than those who had three or fewer injuries.

##### Sociodemographic factors

The relationship between age and injury or illness reporting was inconsistent in two studies [16, 18]. Biddle and Roberts [16] found that older workers were more likely than younger workers to file a WC claim for their injury or illness. In contrast, Siddharthan et al. [18] found a negative association between being older age and injury or illness reporting. The findings on the relationship between gender and injury or illness reporting were also mixed. Two studies [20, 24] found that male workers were less likely than females to report their injuries to their management. Contrary to these findings, Lee et al. [7] and Yang et al. [28] found that female workers were less likely to report their work-related symptoms to management than male workers. Conflicting results were also found for the association between education and reporting [7, 16, 23]. In a study of nursing home workers who had low back pain in the past three months [23], workers with a higher education level were less likely to file a WC claim. On the other hand, among cleaning workers, higher reporting of work-related symptoms to management was associated with a college education [7]. There was a significant association between race/ethnicity and injury or illness reporting [7, 18, 24]. For reporting to management, racial/ethnic minority workers such as Hispanic and Asian were less likely than White workers to report their injury or illness [7, 18]. For reporting to WC, non-White workers were more likely than White workers to file a claim [24]. In addition, lower annual income (less than $40,000) and being married were associated with higher reporting of injury or illness to WC [15, 17].

##### General health and functioning

Three studies [16, 17, 28] investigated the relationship between general health and functioning and injury or illness reporting. There was a positive association between obesity and injury or illness reporting to WC [17]. Workers with better perception of general health were less likely to report their injury or illness to WC and management [16, 28].

##### Worker’s knowledge regarding reporting

Injury or illness reporting was associated with worker’s knowledge regarding reporting [20]. Among 699 surgeons, those who had not heard of reporting experience from their peers were less likely to report their injury or illness to management [20].

##### Job and employment characteristics

Seven studies [16–18, 20, 23, 25, 28] examined the association between job and employment characteristics and injury or illness reporting. Two studies identified work hours as a significant factor for reporting, but the findings were inconsistent. In a study of hospital workers [18], those who worked more than 80 h per two weeks were more likely to report work-related pain. In contrast, in a study of medical residents, longer work hours were associated with underreporting of injury or illness to management [28]. Night shift workers were also less likely than day time workers to report their injury or illness [18]. The following job and employment characteristics were associated with higher reporting to WC programs or management: longer job tenure [15], higher physical demand [16, 23], certain occupations such as nurse, nurse aid, security guard [25], service occupations, precision, craft, and repair occupations, and operators, fabricators, and laborers [17].

##### Psychosocial work environment

Five studies [15, 18, 23, 24, 27] reported a significant association between psychosocial work environment and injury or illness reporting. In five studies [6, 15, 18, 23, 27], a good psychosocial work environment including supervisor support, coworker support, safety training, and safety climate was positively associated with higher injury or illness reporting to WC. Conversely, in a study of patient care workers, an inverse association was identified between organizational policies and safety practices and sharp injury reporting to management [24].

##### Healthcare providers

The type of health care provider was associated with injury or illness reporting [15]. In a study of general workers, those who filed for WC were more likely to receive treatment from a provider not belonging to the company, such as a specialist, surgeon, orthopedic, and physical or occupational therapist [15].

#### Reasons for underreporting

##### Fear or concern

Twelve studies [4, 8, 14, 18, 19, 21, 22, 25–29] reported fear as a reason for underreporting of workers’ injury or illness. Identified barriers to reporting work-related injuries or illnesses included fear of negative consequences on employment status such as missed promotions, job loss, not being hired again [8, 22, 25] or being labeled as a complainer or careless worker and subsequent discrimination [4, 14, 18, 19, 21, 22, 26, 28, 29]. In a focus group study of 28 hospital workers, concern of negative peer attitude was also found as a barrier to reporting [18]. Concerns about administration fortifying safety rules and lack of staffing were identified in two different studies [4, 22].

##### Cumbersome time and effort in reporting process

Ten studies [8, 14, 18, 19, 21, 22, 25–28] identified cumbersome time and effort in the reporting process as a reason for underreporting. In the studies by Weddle et al. [4], Haiduven et al. [14], Moore et al. [22], and Pompeii et al. [25], most workers indicated that they were too busy to report their injuries or illnesses to management. A study of construction workers revealed that workers could not afford to take time off work without payment to see a doctor [22].

##### Lack of knowledge regarding reporting

Nine studies [8, 18, 19, 21, 22, 25–27, 29] identified lack of knowledge of the reporting process as a reason for underreporting. Many workers did not know the official protocols for reporting; for example, how, where, or to whom to report [19, 21, 25, 27]. Studies revealed that some workers did not even know that they should report work-related injury or illness to management [8] and did not receive any training related to reporting [26]. Moreover, many workers did not report because they were uncertain about work-relatedness of their injury or illness [18, 22].

##### Perceptions of injury as not severe or part of the job

Seven studies [4, 14, 22, 25–28] found that minor injury status and perceptions of injury as not severe or part of the job was a reason for underreporting. If the injuries or illnesses were tolerable and sufficiently managed with home treatment, anti-inflammatories, or pain medications, workers perceived their symptoms as minor and chose not to report them [4, 14, 22, 25–28]. The perception that injury is inevitable as part of their job was noted as an obstacle to reporting in two studies [22, 25].

##### Distrust of reporting consequences

Six studies [14, 19, 21, 22, 25, 26] addressed distrust of administrative responses as a barrier to injury reporting. In three studies, some workers pointed out that they perceived no benefits or had no post-event follow-up after injury reporting [14, 21, 25, 26]. Some workers did not trust management in keeping the confidentiality of their reporting [19]. Some workers also reported that, instead of reporting work-related injuries or illnesses to WC, they would choose to get safety incentive benefits for no lost-time injury [22].

### Reporting biases and certainty of evidence

Confidence in the body of evidence is presented in Table 3. The quantitative evidence was rated *very low* for sociodemographic characteristics and general health and functioning and rated *low* for injury type and severity, psychosocial work environment, job and employment, knowledge regarding reporting, and healthcare provider. The quality of outcome was *very low* due to limitations in the study design and sampling methods, inconsistent results, and heterogenous instruments. The qualitative evidence was rated *moderate* for fear, cumbersome time and effort, and perception of injuries as not severe or part of the job and rated *low* for distrust for reporting and lack of knowledge. The quality of outcome was *low* due to the absence of the data related to the concept consisting of both subjective and objective views and the potential of reporting bias.

**Table 3:** The certainty of evidence using GRADE and the criteria support for the concept in the mixed methods synthesis

GRADE				
Contributing factors	Studies	Sample size	Assessment	Explanation
Sociodemographic characteristics				
Age	Biddle [16]	1,598	Low	
Sex	Makary [20], Boden [24], Yang [28], Lee [7]	9.837	Very low	Risk of bias, inconsistency
Race/ethnicity	Boden [24], Lee [7]	1,743	Very low	Risk of bias, inconsistency
Education	Qin [23], Lee [7]	2,810	Very low	Risk of bias, inconsistency
Income	Rosenman [15]	1,598	Low	
Marital status	Fan [17]	321	Very low	Imprecision of results
Injury type and severity				
Activity limitation	Rosenman [15]	1,598	Low	
Absence from work	Rosenman [15]	1,598	Low	
Severe symptoms	Biddle [16], Makary [20], Qin [23]	4,936	Low	Indirectness of evidence
Number of injuries	Makary [20]	699	Very Low	Risk of bias
Psychosocial work environment				
Social support	Rosenman [15], Qin [23]	4,237	Moderate	
Safety training	Lipscomb [6], Boden [24]	2,592	Very low	Risk of bias, indirectness of evidence
Safety incentive	Lipscomb [6]	1,020	Low	Risk of bias
Job strain	Qin [23]	2,639	Low	Risk of bias
Job and employment				
Physical demand	Biddle [16], Qin [23]	4,237	Moderate	
Job tenure	Rosenman [15]	1,598	Low	
Type of work	Fan [17], Makary [20]	1,020	Very low	Risk of bias
Work hour	Yang [28]	7,395	Low	
General health and functioning	Biddle [16], Fan [17], Yang [28]	9,264	Very low	Risk of bias, indirectness of evidence
Knowledge regarding reporting	Makary [20]	699	Low	
Healthcare provider	Rosenman [15]	1,598	Low	
**The criteria of support for the concept in the mixed-methods synthesis**				
Reasons for not reporting	Studies		Assessment	Explanation
Fear	Siddharthan [18], Moore [22], Pompeii [25]		Moderate	Inside-outside
Cumbersome time and effort	Siddharthan [18], Moore [22], Pompeii [25]		Moderate	Inside-outside
Perceptions of injuries as not severe or part of the job	Siddharthan [18], Moore [22], Pompeii [25]		Moderate	Inside-outside
Distrust of reporting	Siddharthan [18], Moore [22], Pompeii [3]		Low	Publication bias, inside-outside
Lack of knowledge	Moore [22], Pompeii [25]		Low	Publication bias, Inside-outside

Risk of bias: limitations in study design and implement; Inconsistency: heterogeneity in study results; Indirectness of evidence: heterogeneity in measurement tools used or operationalization of outcome; Imprecision of results: wide confidence intervals and small sample size; Truth value/bias: the inferences related to an analytical concept remain sensitive to, and clearly reflective of the numeric and textual data from the primary studies; Explanation credibility: the analytical concept and the related inferences are theoretically and conceptually sound; Weakness minimization: the concept is supported by a range of data from different study designs; Inside-outside: the data related to the concept consists of both subjective (insider) views and objective (outsider) observations; Publication bias: there is at least one study that shows non-significant, null, or contrasting results

## Discussion

The purpose of this systematic review was to estimate the prevalence of underreporting of work-related injuries or illnesses and to identify contributing factors and reasons for underreporting. The review of the eligible 20 studies showed that a substantial number of workers who experienced work-related injuries or illnesses (20–91%) did not report their symptoms to management or WC programs. We identified the following contributing factors to injury or illness underreporting: injury type and severity, sociodemographic factors, general health and functioning, worker’s knowledge about reporting, job and employment characteristics, psychosocial work environment, and healthcare providers. Consistent with a previous study by Azaroff et al. [32], underreporting was higher among racial/ethnic minority workers, those with lower income, and workers in poor psychosocial work environments. The relationships of age, gender, educational levels, work hour, and safety climate to underreporting were inconsistent across studies. Our findings are also in line with findings of Pransky et al.’s study [33], which identified the following as reasons for underreporting: fear or concern, cumbersome time and effort in the reporting process, lack of knowledge regarding reporting, perceptions of injuries as not severe or part of the job, and distrust of reporting consequences.

### Methodological limitations of included studies

Measurement of work-related injuries or illnesses is important information to compare and synthesize results from different studies on the prevalence, contributing factors, and reasons for workers’ underreporting of injuries or illnesses. This review identified that the measurements of work-related injuries or illnesses varied across studies and were not always denoted. The constitution of reportable injuries may vary by company ranging from including near misses and unsafe conditions to only actual injuries [34]. In regard to WC systems, the eligibility for WC benefits for medical treatment is consistent across states in the US, but the length of time that workers can receive temporary disability benefits for lost workdays differs from state to state [35]. Biddle and Roberts [16] and Rosenman et al. [15] used multiple approaches to measure underreporting rates for WC medical benefits and temporary disability benefits. On the other hand, Scherzer et al. [8], Fan et al. [17], Qin et al. [23], Anderson et al. [29], and Green et al. [27] measured underreporting in a relatively broad extent of work-related injuries or illnesses. All of the studies except for Fan et al. [17] measured reporting of any work-related injury or illness without requiring a confirmed diagnosis by a physician. It is also important to note that the timeframe used to measure the prevalence of underreporting varied across studies. The prevalence of underreporting was lowest in Lipscomb et al.’s study measuring lifetime prevalence [6] and the highest in Qin et al.’s study measuring with three-month prevalence [23], which may be due to recall bias. If a timeframe of reporting used in the survey question was too short, the reporting experience of workers may not have been fully captured. Conversely, if a time frame was too long, workers may have only remembered severe injury events. The differences in measurement of underreporting of work-related injury or illness using different timeframes across studies interfere with comparisons of study findings.

### Strengths and limitations of the current review

The present study is one of the first systematic reviews that investigated workers’ underreporting of occupational injury or illness in the United States. Including both quantitative and qualitative design in the review strengthens the review findings. This review has several limitations. First, this review used only five electronic databases and included only English publications. Therefore, this review may have not fully captured all relevant studies. Second, this review has limited generalizability due to heterogeneity of included sample characteristics and study setting, study design, low certainty of overall evidence, and potential publication bias toward studies. Last, although revised OSHA 2014 reporting regulations may have influenced on workers’ reporting behaviors, we identified and included only eight studies that were published after 2014; however, there was no big difference of the findings between studies published before and after 2014.

### Implications for future research

This systematic review demonstrates that various factors have affected the reporting of work-related injuries or illnesses among workers and there are many challenges to adequately measuring the level of underreporting. To accurately and fully capture all work-related injuries and illnesses, it is important to minimize barriers that workers can experience in the process of reporting their work-related injuries or illnesses. In addition, an objective measurement for underreporting of work-related injury or illness is required. However, we found an absence of a standardized approach to measuring injury or illness reporting, and this resulted in a wide variation in the measured prevalence of underreporting across studies. In the present review, most of included studies employed a cross-sectional study design, which limited the ability to determine causal relationship between various factors noted above and workers’ underreporting of injury or illness. All these findings highlight the need for future research employing a longitudinal study design and standardized measurement of workers’ underreporting.

## Conclusions

Our review findings show that the level of underreporting of work-related injury or illness varies by use of different measurement approaches. Nevertheless, underreporting of work-related injury or illness was found to be common among workers, particularly among vulnerable groups such as racial/ethnic minorities with low wages and poor psychosocial work environments. Our findings can give insights for employers and public health administrators into improving organizational safety culture and climate, and for empowering these vulnerable groups regarding work-related injury or illness reporting. Future research applying a standardized measurement and longitudinal study design can provide strong evidence for the development of interventions to eliminate the barriers to reporting work-related injuries or illnesses.

### Registration and protocol

This review was reported based on the Preferred Reporting Items for Systematic Reviews and Meta-Analyses (PRISMA) guidelines [36]. The review was registered in the PROSPERO, an international database of prospectively registered systematic reviews in health and social care (CRD42021284685).

## Supplementary Information

**Additional file 1.**

## Data Availability

All data generated or analyzed during this study are included in this published article.

## Abbreviations

WC: Worker’s compensation
BLS: Bureau of Labor Statistics
PRISMA: Preferred Reporting Items for Systematic Reviews and Meta-Analyses
OSHA: Occupational Safety and Health Administration
MeSH: Medical Subject Headings
