# Supplementary material for: Underreporting of workers’ injuries or illnesses and contributing factors: a systematic review
Source: BMC Public Health. 2023 Mar 24;23:558. doi: 10.1186/s12889-023-15487-0 (PMC10037763; doi:10.1186/s12889-023-15487-0)
Supplement: Supplementary file 1 — Additional file 1. [file 12889_2023_15487_MOESM1_ESM.docx]

**Supplementary Materials**

Appendix 1. Search strategy.

**PubMed via MEDLINE** (Search date: November 10, 2022; 1140 results. post deduplication 1,138)

| # | Searches | Results |
| --- | --- | --- |
| 1 | ("Accidents, Occupational"[MeSH]) OR ("Occupational Diseases"[MeSH]) OR ("Occupational Injuries"[MeSH]) OR ("occupational disease*"[Title/Abstract]) OR ("occupational injur*"[Title/Abstract]) OR ("work related illness*"[Title/Abstract]) Filters: **English** | 158,215 |
| 2 | ("Mandatory Reporting"[MeSH]) OR ("underreport*"[Title/Abstract]) OR ("under-report*"[Title/Abstract]) OR ("underestimat*"[Title/Abstract]) OR ("under-estimat*"[Title/Abstract]) OR ("undercount*"[Title/Abstract]) OR ("under-count*"[Title/Abstract]) OR ("underrecord*"[Title/Abstract]) OR ("under-record*"[Title/Abstract]) Filters: **English** | 95,099 |
| 3 | (("Accidents, Occupational"[MeSH]) OR ("Occupational Diseases"[MeSH]) OR ("Occupational Injuries"[MeSH]) OR ("occupational disease*"[Title/Abstract]) OR ("occupational injur*"[Title/Abstract]) OR ("work related illness*"[Title/Abstract])) AND (("Mandatory Reporting"[MeSH]) OR ("underreport*"[Title/Abstract]) OR ("under-report*"[Title/Abstract]) OR ("underestimat*"[Title/Abstract]) OR ("under-estimat*"[Title/Abstract]) OR ("undercount*"[Title/Abstract]) OR ("under-count*"[Title/Abstract]) OR ("underrecord*"[Title/Abstract]) OR ("under-record*"[Title/Abstract])) Filters: **English** | 1,140 |

**PsycINFO via ProQuest** (Search date: November 13, 2022; 200 results. post deduplication 200)

| # | Searches | Results |
| --- | --- | --- |
| 1 | subject(industrial accident) OR subject(work related illness) OR (accidents, occupational) OR (occupational disease*) OR (occupational injur*) Filters: **English** | 24,512 |
| 2 | subject(mandatory report*) OR underreport* OR under-report* OR underrecord* OR under-record* OR underestimat* OR under-estimat* OR undercount* OR under-count* Filters: **English** | 17,619 |
| 3 | (((accidents, occupational) OR subject(industrial accident) OR subject(work related illness) OR (occupational disease*) OR (occupational injur*)) AND la.exact("ENG")) AND (subject(mandatory report*) OR underreport* OR under-report* OR underrecord* OR under-record* OR underestimat* OR under-estimat* OR undercount* OR under-count*) | 200 |

**CINAHL via EBSCO (**Search date: November 14, 2022; 192 results. post deduplication 180)

| # | Searches | Results |
| --- | --- | --- |
| 1 | (MH accidents, occupational OR occupational disease* OR occupational injur* OR work related illness*) AND **English** (Languages) | 15,541 |
| 2 | (MH mandatory reporting OR underreport* OR under-report* OR underrecord* OR under-record* OR undercount* OR under-count* OR underestimate* OR under-estimat*) AND **English** (Languages) | 27,788 |
| 3 | 1 AND 2 | 192 |

**Embase via Embase.com (**Search date: November 11, 2022; 1,352 results. post deduplication 1.349)

| # | Searches | Results |
| --- | --- | --- |
| 1 | (('occupational accident'/exp) OR ('occupational disease'/exp) OR ('occupational injury'/exp) OR ('occupational accident') OR ('occupational disease'))AND [english]/lim | 226,089 |
| 2 | (('mandatory reporting'/exp) OR ('under reporting'/exp) OR (under reporting) OR (undercount)) AND [english]/lim | 13,636 |
| 3 | 1 AND 2 | 1,352 |

**Social Science Citation Index via Web of Science (**Search date: November 13, 2022; 287 results. post deduplication 254)

| # | Searches | Results |
| --- | --- | --- |
| 1 | TS=(occupational accident* OR occupational injur* OR occupational disease* OR work related illness*) AND **English** (Languages) | 17,094 |
| 2 | TS=(mandatory report* OR underreport* OR underestimat* OR undercount* OR underrecord* Or under-report* OR under-count* OR OR under-estimat* OR under-record*) AND **English** (Languages) | 26,983 |
| 3 | #1 AND #2 | 287 |

**Appendix 2** MMAT quality appraisal profile.

| Screening | S1. Are there clear research questions or objectives? | | | | |
| --- | --- | --- | --- | --- | --- |
|  | S2. Do the collected data allow the research questions or objective to be addressed? | | | | |
|  | **RANDOMIZED CONTROLLED TRIALS** | | | | |
| Author, year | Is randomization appropriately preformed? | Are the groups comparable at baseline? | Are there complete outcome data? | Are outcome assessors blinded to the intervention provided? | Did the participants adhere to the assigned intervention? |
| Green,  2019 [16] | Yes | Yes | Yes | Can’t tell | Yes |
|  | **NON-RANDOMIZED STUDIES** | | | | |
| Author, year | Are the participants representative of the target population? | Are measurements appropriate regarding both the outcome and intervention (or exposure)? | Are there complete outcome data? | Are the confounders accounted for in the design and analysis? | During the study period, is the intervention administered (or exposure occurred as intended? |
| Rosenman,  2000 [17] | Yes | Yes | Yes | Yes | Yes |
| Biddle,  2003 [18] | Yes | Yes | Yes | No | Yes |
| Fan,  2006 [19] | Yes | Yes | Yes | Yes | Yes |
| Makary,  2007 [20] | No | Yes | Yes | Yes | Yes |
| Lipscomb,  2013 [6] | No | Yes | Yes | Yes | Yes |
| Qin,  2014 [21] | No | Yes | Yes | Yes | Yes |
| Boden,  2015 [22] | No | Yes | Yes | No | Yes |
| Yang,  2019 [23] | Yes | Yes | Yes | Yes | Yes |
| Lee,  2021 [7] | No | Yes | Yes | Yes | Yes |
|  | **QUANTITATIVE DESCRIPTIVE STUDIES** | | | | |
| Author, year | Is the sampling strategy relevant to address the research question? | In the sample representative of the target population? | Are the measurements appropriate? | Is the risk of nonresponse bias low? | Is the statistical analysis appropriate to answer the research question? |
| Weddle,  1996 [4] | Yes | No | No | No | Yes |
| Haiduven,  1999 [24] | Yes | No | Yes | Can’t tell | Yes |
| Scherzer,  2005 [8] | Yes | No | Yes | Yes | Yes |
| Gershon,  2007 [25] | Yes | Yes | Yes | No | Yes |
| Donnelly,  2013 [26] | Yes | No | Yes | No | Yes |
| Deipolyi,  2017 [27] | Yes | No | Yes | No | Yes |
| Anderson,  2021 [28] | Yes | No | Yes | No | Yes |
|  | **MIXED METHOD STUDIES** | | | | |
| Author, year | Is there an adequate rational for using a mixed methods design to address the research question? | Are the different components of the study effectively integrated to answer the research question? | Are the outputs of the integration of qualitative and quantitative components adequately interpreted? | Are divergences and inconsistencies between quantitative and qualitative results adequately addressed? | Do the different components of the study adhere to the quality criteria of each tradition of the methods involved? |
| Siddharthan,  2006 [29] | Yes | Yes | No | No | No |
| Moore,  2013 [30] | No | Yes | Yes | No | No |
| Pompeii,  2015 [31] | No | Yes | Yes | Yes | No |
